# Supplementary material for: Digital Competence as Psychological Defense: Impact of Digital Competence on Problematic Mobile Use Among Paraguayan University Students
Source: Behav Sci (Basel). 2025 Dec 5;15(12):1687. doi: 10.3390/bs15121687 (PMC12729974; doi:10.3390/bs15121687)
Supplement: Supplementary file 1 [file behavsci-15-01687-s001.zip › behavsci-3955389-supplementary.pdf]

Table S1. Summary statistics of key study variables (N = 500).

Variable	Mean	SD	Min	Max
Age (years)	21.4	2.7	18	29
Digital Competence – Total (DCQ-US)	4.1	0.5	1.0	5.0
├─ Information Literacy	4.2	0.6	1.0	5.0
├─ Communication & Collaboration	4.0	0.6	1.0	5.0
├─ Content Creation	3.9	0.7	1.0	5.0
├─ Safety & Privacy	4.3	0.5	1.0	5.0
├─ Problem Solving	4.1	0.6	1.0	5.0
Problematic Smartphone Use – Total (PMPUQ-R)	2.9	0.6	1.0	4.0
├─ Dependent Use	3.1	0.7	1.0	4.0
├─ Prohibited Use	2.8	0.6	1.0	4.0
├─ Dangerous Use	2.7	0.6	1.0	4.0

Gender distribution: 61% female (coded as 0), 39% male (coded as 1).

Behavioral Indicators (Prevalence)

Item	Yes (%)
Frequent checking of notifications	84%
Extended smartphone use beyond intended time	71%
Social media use during study time	69%
Multitasking with non-academic apps	63%
Anxiety when phone is unavailable	72%

Correlation Supporting the Scatterplots

- r (DCQ-US Total × PMPUQ-R Total) = −0.38, $p < 0.001$

  Note: All values are aggregated to ensure participant anonymity.
